# Two-year outcomes of trabeculo-canalectomy for glaucoma secondary to iridocorneal endothelial syndrome: a retrospective study

**DOI:** 10.3389/fmed.2026.1883138

**Published:** 2026-07-15

**Authors:** Zhan Xie, Haiyue Xie, Yangyang Lu, YingTing Zhu, Ping Xie, Hong Sun

**Affiliations:** 1 Department of Ophthalmology, The First Affiliated Hospital with Nanjing Medical University, Nanjing, Jiangsu Province, China; 2 R&D Department, BioTissue, Miami, FL, United States

**Keywords:** glaucoma secondary to iridocorneal endothelial syndrome, inner aqueous drain, IOP, trabeculo-canalectomy, two-year outcomes

## Abstract

**Background:**

The study aimed to evaluate the 2-year outcomes of a new surgical procedure for treating Chinese patients with glaucoma secondary to iridocorneal endothelial syndrome (GS-ICE).

**Methods:**

This was a retrospective study involving 26 GS-ICE patients (26 eyes) surgically treated with trabeculo-canalectomy in the First Affiliated Hospital with Nanjing Medical University from January 2015 to June 2024, and postoperatively followed up for 2 years. An intraocular pressure (IOP) of lower than 21 mmHg (1 mmHg = 0.133 kPa) with (conditional success) or without anti-glaucoma medication (complete success) was set as the cut-off value of surgical success. Main outcomes of trabeculo-canalectomy for GS-ICE included IOP, number of medications, surgical success, and complications.

**Results:**

A total of 26 GS-ICE patients (26 eyes) with an average age of 48.58 ± 9.61 (27–67) years and a median number of 2 (0–4) anti-glaucoma medication regimens were retrospectively included. Compared to the preoperative value (31.10 ± 11.00 mmHg), the postoperative mean IOP was significantly lower, reaching 13.27 ± 2.68, 15.00 ± 3.77, 15.04 ± 2.81, 16.27 ± 3.03, 16.96 ± 3.12, and 16.89 ± 3.18 mmHg at 1 week, 1 month, 3 months, 6 months, 1 year, and 2 years, respectively (*p* < 0.001). The number of postoperative anti-glaucoma medications was also significantly reduced to 0 (0–0), 0 (0–2), 0 (0–1), 0 (0–2), 0 (0–2), and 0 (0–2) at the six follow-up visits, respectively (*p* < 0.001). The complete and conditional success of trabeculo-canalectomy in treating GS-ICE at 2 years was 80.8% and 11.5%, respectively. Hyphema (7.7%), the most common early-stage complication, was self-relieved during the first week postoperatively. Two cases developed corneal edema without elevated IOP, and two with surgical failures were treated successfully with an Ahmed valve implantation.

**Conclusion:**

Trabeculo-canalectomy brings favorable two-year outcomes among GS-ICE patients, including an effective IOP control, a reduction of anti-glaucoma medications, and a low risk of postoperative complications.

## 1 Introduction

Iridocorneal endothelial (ICE) syndrome, a rare ophthalmic disorder, is caused by over-proliferated corneal endothelial cells migrating across the iridocorneal angle and onto the iris, frequently leading to peripheral anterior synechiae (PAS) formation and intraocular pressure (IOP) elevation (1). Secondary glaucoma is a common complication affecting 46–82% of patients with ICE syndrome (2).

Generally, medications are preferred to glaucoma secondary to iridocorneal endothelial syndrome (GS-ICE) in the early stage. Nearly half of GS-ICE patients, however, suffer from a failure of medications and require surgical treatment with trabeculectomy and glaucoma drainage device (GDD) implantation (3). Trabeculectomy for GS-ICE usually ends in a poor surgical outcome due to endothelial cell proliferation, onset at a younger age, and long-term scarring of filtering blebs (4, 5). Though GDD implantation is more efficient in controlling IOP than trabeculectomy, its long-term clinical outcomes can be influenced by endothelial damages caused by the drainage tube and tube exposure due to improper coverage with a patch graft (5). Additionally, postoperative IOP control is a big challenge. The majority of GS-ICE patients require multiple anti-glaucoma surgeries to control IOP (6). As a result, GS-ICE is often refractory (7), highlighting the urgent need to improve the existing surgical treatment.

Similar to most other secondary angle-closure glaucoma, the obstructed pre-trabecular meshwork (TM) in ICE syndrome leads to the secondary angle-closure glaucoma, but the outflow system is often intact (5). Therefore, we propose a surgical plan to establish a direct channel between the anterior or posterior chamber to the Schlemm’s canal (SC), thereby effectively controlling the postoperative IOP in angle-closure GS-ICE eyes. Our previous study has demonstrated the high efficacy of a bleb-independent trabeculo-canalectomy in treating primary angle-closure glaucoma (PACG) (8). ICE, a subtype of secondary angle-closure glaucoma (5), may also benefit from this surgical procedure. In this retrospective study, we reported the two-year outcomes of trabeculo-canalectomy for treating Chinese patients with GS-ICE.

## 2 Methods

### 2.1 Study and participants

This was a retrospective study following the *Declaration of Helsinki*, and approved by the Ethics Committee of the First Affiliated Hospital with Nanjing Medical University (2023-SR-474). All participants presented informed consent preoperatively. From January 2015 to June 2024, patients with ICE syndrome who were surgically treated with trabeculo-canalectomy in the First Affiliated Hospital with Nanjing Medical University and followed up for 2 years were retrospectively included. A diagnosis of ICE syndrome was made according to positive findings of a unilateral hammered-silver appearance of the posterior corneal surface, corneal edema, iris stroma abnormalities, and ICE cells, regardless of iris nodules and widespread PAS (9, 10). Patients with ICE syndrome were further classified into three subtypes: Chandler’s syndrome (corneal edema with minimal iris stromal atrophy), essential iris atrophy (pronounced stromal atrophy and hole formation in the iris), and Cogan–Reese syndrome (nodules on the iris with varying degrees of atrophy) (10, 11). Only patients with ICE syndrome subtype of essential iris atrophy were included in this study. Individuals with previous eye surgeries or ocular trauma that might disrupt the integrity of SC were excluded.

### 2.2 Surgical procedures of trabeculo-canalectomy

**Figure 1:**
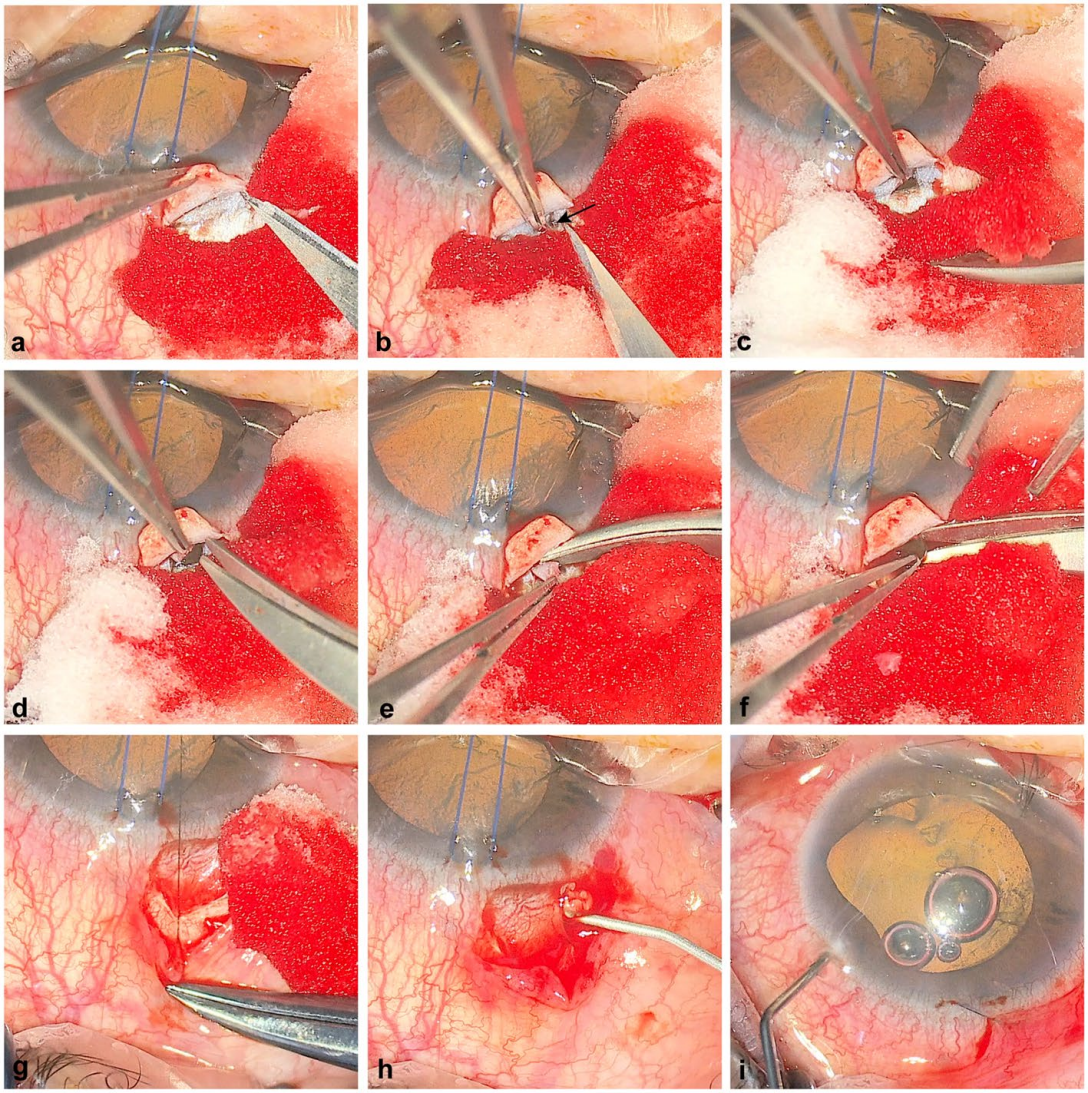
Surgical procedures of trabeculo-canalectomy. **(a)** A 3 × 4 mm, half-thickness, rectangular superficial scleral flap dissected at the superior limbus into the transparent cornea. **(b)** Localization of the SC. The broken end of SC pointed by black arrows. **(c)** An opening made on the outer wall of SC. **(d)** A small incision on the iris using corneal scissors. **(e)** Removal of the outer wall of SC and the juxtacanalicular TM. **(f)** Peripheral iridectomy. **(g)** Two 10-0 nylon sutures for tightly closing the scleral flap. **(h)** Iris repositioning by injecting a moderate volume of viscoelastic agents under the scleral flap. **(i)** Removal of residual viscoelastic agents by irrigating the anterior chamber.

All surgical procedures of trabeculo-canalectomy without the use of antimetabolites were operated by the same experienced surgeon as previously described (8, 12). Briefly, a 2.5-mL mixture (1:1) of lidocaine 2% and bupivacaine 0.75% was administered for peribulbar anesthesia. Following the preparation of an 8-0 polypropylene suture in the corneal limbus, a fornix-based conjunctival trabeculectomy flap was made on the Tenon’s capsule. Then, a 3 × 4 mm, half-thickness, superficial scleral flap was dissected (Figure 1a). Next, the clear corneal area and the white sclera area were identified. The gray zone, also known as the grayish blue trabecular meshwork, was delineated. The Schlemm’s canal runs at the junction between the grayish blue trabecular meshwork and the white sclera, and the Schlemm tube was accurately located at the posterior 1/2 of the gray area (8). After localizing the SC (Figure 1b), its outer wall was exposed and opened to restore the aqueous humor outflow (Figure 1c). A small incision was made on the iris with corneal scissors (Figure 1d) prior to the removal of the outer wall of SC (2 mm in length) and the adjacent TM (Figure 1e). Following peripheral iridectomy (Figure 1f), two 10-0 nylon sutures were made to tightly secure the scleral flap (Figure 1g). The iris was re-positioned by injecting a moderate volume of viscoelastic agents under the scleral flap (Figure 1h). The conjunctiva-Tenon’s layer was fused with 10-0 nylon sutures at bilateral incisional cuts. Finally, a side incision was made on the cornea, and the anterior chamber was irrigated to remove the residual viscoelastic agents (Figure 1i).

### 2.3 Follow-up visits

IOP, number of anti-glaucoma medications, best corrected visual acuity (BCVA), anterior/posterior segment, complications and interventions, and status of filtering blebs were postoperatively followed up at 1 week, 1 month, 3 months, 6 months, 1 year, and 2 years.

### 2.4 Assessment of surgical success and failure of trabeculo-canalectomy

Surgical success of trabeculo-canalectomy was defined as postoperative IOP ranging from 6 to 21 mmHg (1 mmHg = 0.133 kPa) with (conditional success) or without anti-glaucoma medications (complete success). A poor control of postoperative IOP (<6 mmHg or >21 mmHg) even after medicating with anti-glaucoma drugs was considered as a surgical failure (8, 12). In these cases, severe complications, like retinal detachment and endophthalmitis, were investigated.

The Indiana Bleb Appearance Grading Scale (IBAGS) classifies the height and extent of filtering blebs into four grades. The height of filtering blebs is graded into H0-3 according to the elevation of the conjunctival flap vertically above the scleral surface: H0, none; H1, mild; H2, moderate; and H3, significant elevation. The extent of filtering blebs, defined as the horizontal dimension or the coverage area, is categorized into E0 (no visible or <1 clock hour), E1 (1–2 clock hours), E2 (2–4 clock hours), and E3 (>4 clock hours).

### 2.5 Statistical analysis

Statistical analyses were processed using SPSS 21.0. Continuous variables, expressed as mean ± standard deviation or median (range), were analyzed by the *t*-test and the non-parametric test for samples in normal and skew distributions, respectively. Categorical variables were represented as case number and percentage. Postoperative IOP values and mean deviation (MD) of visual field (VF) at different follow-up time points were compared by the repeated measures analysis of variance (ANOVA). Chi-square analysis was used to analyze the drug use before and after surgery. Friedman test was used to analyze the Logmar BCVA before and after surgery. The cumulative surgical success of trabeculo-canalectomy in treating GS-ICE was measured by the Kaplan-Meier estimate. *p* < 0.05 was considered as a significant difference.

## 3 Results

In this retrospective study, 26 GS-ICE patients (26 eyes) with a mean age of 48.58 ± 9.61 (27–67) years, an average preoperative IOP of 31.10 ± 11.00 mmHg, and a median preoperative number of medications of 2 (1–4) were included, involving 8 (30.77%) men and 18 (69.23%) women (Table 1). These patients had the ICE syndrome subtype of essential iris atrophy and all operated eyes were phakic.

**Table 1:** Characteristics of GS-ICE patients enrolled.

Characteristics	Values
Case number/eyes (*n*)	26/26
OD/OS (*n*)	12/14
Han Chinese (*n*, %)	26 (100%)
Male/female (*n*)	8/18
Age (years)	48.58 ± 9.61
Preoperative MD (dB)	−16.38 ± 4.44
Preoperative IOP (mmHg)	31.10 ± 11.00
Preoperative medications (*n*)	2.31 ± 0.68
Preoperative BCVA (Logmar)	0.21 ± 0.08

GS-ICE, glaucoma secondary to iridocorneal endothelial syndrome; OD, oculus dexter; OS, oculus sinister; MD, mean deviation; IOP, intraocular pressure; BCVA, best corrected visual acuity; Logmar, logarithm of the minimum angle of resolution; ICE, iridocorneal endothelial.

**Figure 2:**
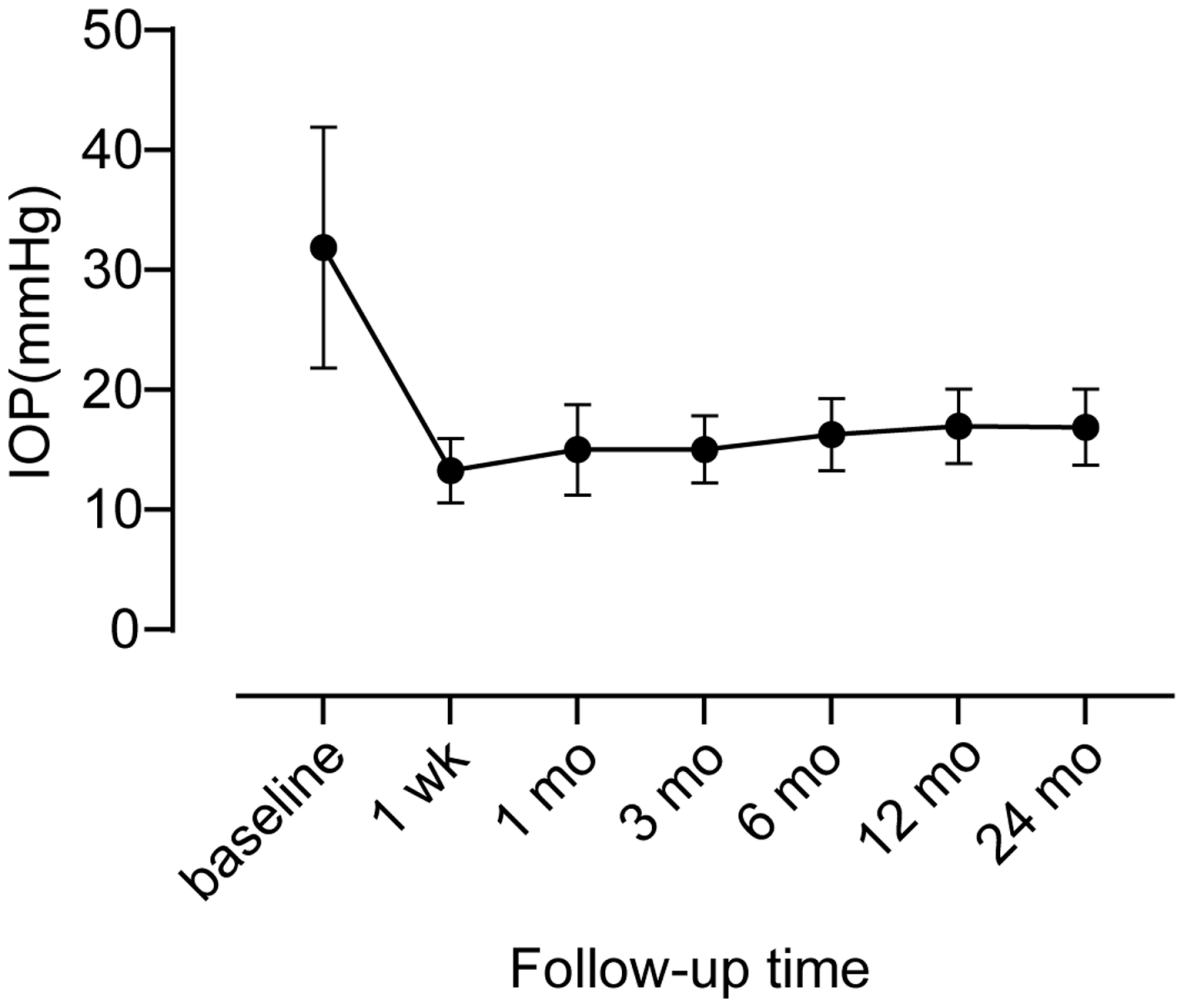
IOP measurements at baseline and postoperative follow-up visits. IOP values were shown as mean ± SD. IOP, intraocular pressure; SD, standard deviation.

Compared to the preoperative value (31.10 ± 11.00 mmHg), the postoperative mean IOP at different follow-up time points was significantly lower, reaching 13.27 ± 2.68, 15.00 ± 3.77, 15.04 ± 2.81, 16.27 ± 3.03, 16.96 ± 3.12, and 16.89 ± 3.18 mmHg at 1 week, 1 month, 3 months, 6 months, 1 year, and 2 years, respectively (all *p* < 0.001) (Figure 2). Notably, none of the affected eyes required medications at 1 week postoperatively. The proportion of medicated eyes at each follow-up visit was significantly reduced (*p* < 0.001) (Table 2). The number of postoperative anti-glaucoma medications was also significantly reduced to 0 (0–0), 0 (0–2), 0 (0–1), 0 (0–2), 0 (0–2), and 0 (0–2) at the six follow-up visits (all *p* < 0.001) (Table 2). The median (range) of Logmar BCVA were 0.2 (0.1–0.6), 0.2 (0.1–0.3), 0.2 (0.1–0.3), 0.2 (0.1–0.3), 0.2 (0–0.3), 0.2 (0–0.3), 0.3 (0–0.4) at the seven different time points, which showed significantly difference (*p* < 0.001) (Table 2). Compared to the preoperative MD of VF (−16.38 ± 4.44 dB), the postoperative mean MD at different follow-up time points showed no significant difference, reaching 16.53 ± 4.22 and 16.70 ± 4.70 dB at 1 year, and 2 years, respectively (*p* = 0.966).

**Table 2:** Two-year outcomes of a trabeculo-canalectomy for treating GS-ICE.

Follow-up time point	IOP (mmHg)	Medicated eyes (*n*, %)	Number of medications	Visual acuity (Logmar)
Baseline	31.10 ± 11.00	26 (100)	2 (1–4)	0.2 (0.1–0.6)
1 week	13.27 ± 2.68	0	0 (0–0)	0.2 (0.1–0.3)
1 month	15.00 ± 3.77	2 (7.7)	0 (0–2)	0.2 (0.1–0.3)
3 months	15.04 ± 2.81	2 (7.7)	0 (0–1)	0.2 (0.1–0.3)
6 months	16.27 ± 3.03	2 (7.7)	0 (0–2)	0.2 (0–0.3)
1 year	16.96 ± 3.12	3 (11.5)	0 (0–2)	0.2 (0–0.3)
2 years	16.89 ± 3.18	5 (19.2)	0 (0–2)	0.3 (0–0.4)
*p*-value	<0.001^*^	<0.001^†^		<0.001^#^

IOP data were expressed as mean ± SD, Medications and visual acuity data were expressed as median (range).

GS-ICE, glaucoma secondary to iridocorneal endothelial syndrome; IOP, intraocular pressure; SD, standard deviation.

^*^Data were analyzed by the one-way ANOVA.

^†^Data were analyzed by the Chi-square test.

^#^Data were analyzed by the Friedman test.

**Figure 3:**
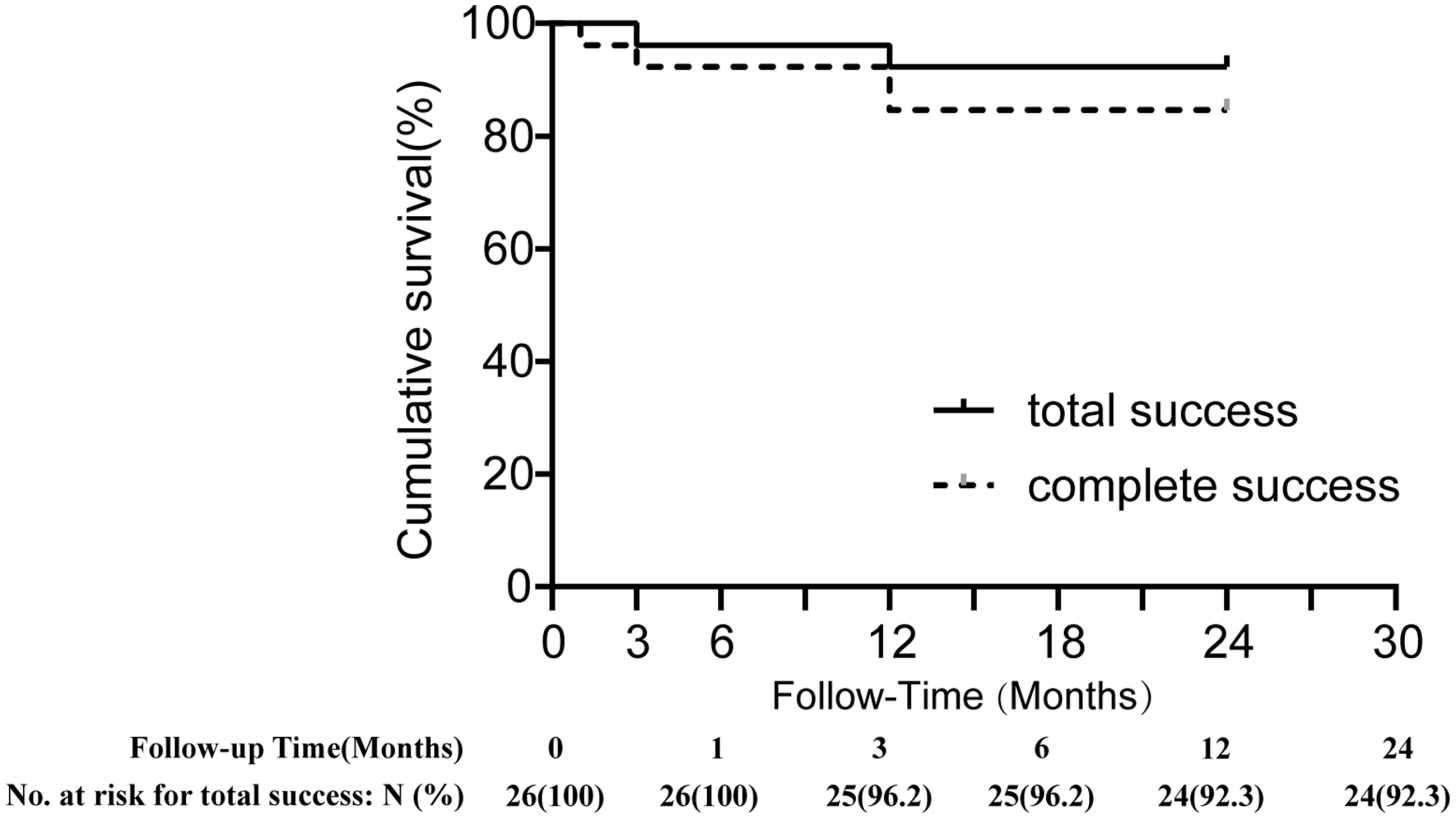
Kaplan-Meier plots of the cumulative surgical success of trabeculo-canalectomy in treating GS-ICE.

At 2 years of follow-up, the overall surgical success of trabeculo-canalectomy in treating GS-ICE was 92.3%, with the complete success of 80.8% and the conditional success of 11.5% (Table 3 and Figure 3). Only one filtering bleb in one (3.8%, 1/26) eye was graded as E1H1 at 1 week, and filtering blebs in all treated eyes (100%, 26/26) were classified into E0H0 at 1 month. Hyphema (7.7%), the most common early-stage complication, was self-relieved during the first week postoperatively. Two cases had uncontrolled IOP on maximal anti-glaucoma medications and were treated successfully with an Ahmed valve implantation at postoperative 13 months. Two cases developed corneal edema without elevated IOP 2 years after surgery. Malignant glaucoma, hypotony, blebitis, endophthalmitis, and choroidal detachment were not noted during the follow-up period.

**Table 3:** Surgical success of trabeculo-canalectomy in treating GS-ICE.

Follow-up time point	Total success (*n*, %)	Complete success (*n*, %)	Conditional success (*n*, %)
1 week	26 (100)	26 (100)	0
1 month	26 (100)	25 (96.2)	1 (3.8)
3 months	25 (96.2)	24 (92.3)	1 (3.8)
6 months	25 (96.2)	24 (92.3)	1 (3.8)
1 year	24 (92.3)	22 (84.6)	2 (7.7)
2 years	24 (92.3)	21 (80.8)	3 (11.5)

GS-ICE, glaucoma secondary to iridocorneal endothelial syndrome.

## 4 Discussion

Though various filtrating surgeries have been applied in patients with GS-ICE, but its long-term outcomes may be largely challenged by sub-conjunctival fibrosis and abnormal bleb structures (3). Trabeculectomy using antimetabolites, a representative filtering procedure, shows an acceptable intermediate-term outcome in treating GS-ICE. In comparison to juvenile, pigmentary, and primary open-angle glaucoma, the long-term efficacy of trabeculectomy in treating GS-ICE, however, is less favorable due to a pathological proliferation of endothelium and structural defects in the bleb (13, 14).

In the present study, we, for the first time, reported the efficacy of trabeculo-canalectomy, a novel modified surgical procedure of trabeculectomy with an internal route of drainage, in the treatment of GS-ICE, yielding an overall success of 92.3% and a complete success of 80.8% at 2 years postoperatively (Table 3 and Figure 3). The surgical success of trabeculo-canalectomy in controlling postoperative IOP among GS-ICE patients, regardless of continued anti-glaucoma medications, was significantly higher than the previously reported statistics (Table 4) (6, 11, 13, 15–19). Hyphema (7.7%) was the predominant postoperative complication, but self-relieved within the first week. Collectively, we validated the high long-term efficacy and safety of trabeculo-canalectomy in treating Chinese patients with GS-ICE.

**Table 4:** Current study on the 2-year surgical success in treating GS-ICE.

Author, year reference	Surgical procedure (sample size)	Success criteria	2-year total success rate	2-year complete success rate
Nilforushan N et al., 2024 (6)	Trab (*n* = 13) vs. AGV (*n* = 16)	Complete success was defined according to the level of IOP (≤18) and at least 20% reduction from preoperative IOP without AGMs. Qualified success was defined as complete success with medications, where the number of medications was less than preoperative numbers.	Trab 84.6% AGV 82.25%	Trab 30.8% AVG 18.75%
Senthilkumar VA et al., 2023 (11)	AADI (*n* = 21) vs. Trab + MMC (*n* = 20)	Surgical failure was defined as IOP > 21 mmHg or reduced <20% from baseline, IOP ≤ 5 mmHg, reoperation for glaucoma or a complication, or loss of light perception vision.	AADI 76% Trab 50%	AADI 57% Trab 45%
Gebremichael BG et al., 2020 (15)	AGV (*n* = 18)	Surgical success was defined as IOP ≥ 6 and ≤21 mm Hg with or without AGMs.	83.1%	N/A
Mao Z et al., 2021 (16)	AGV (*n* = 18)	The procedure was considered a complete success if the long-term IOP was ≤21 mmHg without AGM or further filtration surgery for the entire course of available follow-up, a qualified success if the IOP < 21 mmHg was obtained with AGMs.	88.1%	N/A
Chandran P et al., 2016 (13)	Trab + MMC (*n* = 16)	Surgical success was defined as complete when the IOP was ≥5 and ≤21 mm Hg with no additional AGMs and as qualified if IOP was ≤21 mm Hg with or without AGMs.	82% at 1 year,71% at 3 years	64% at 1 year, 57% at 3 years
Doe EA et al., 2001 (17)	Trab (*n* = 5), Trab + GDI (*n* = 7) vs. GDI (*n* = 14)	Success was defined as a postoperative IOP of 21 mmHg or less with or without AGMs and vision of at least 20/400 (severe visual impairment or better) during the follow-up period. Success included manipulation of a prior glaucoma site such as bleb needling, needling over a GDI plate, or neodymium:yytrium aluminum–garnet laser to the tube tip or filter ostium.	Trab: 73% at 1 year, 44% at 3 years, 29% at 5 years GDI: 71% at 1 year, 71% at 3 years, and 53% at 5 years.	N/A
Lanzl IM et al., 2000 (18)	Trab + MMC (*n* = 10)	Adequate control of IOP (without medication lower than 21 mmHg).	N/A	80% at the last follow-up (follow-up time: 9–19 months)
Kim DK et al., 1999 (19)	Aqueous shunt surgery (*n* = 10)	Complete success was defined as a postoperative IOP of 21 mmHg or lower, with or without AGMs, for the entire course of available follow-up. Qualified success was defined as IOP of 21 mmHg or lower, with or without AGMs, at most recent follow-up in eyes that had undergone one or more tube repositions or revisions of the initial aqueous shunt surgery during available follow-up.	70% at 1 year, 40% at 5 years	N/A

Trab, trabeculectomy; AGV, Ahmed glaucoma valve; AGMs, anti-glaucoma medications; AADI, Aurolab aqueous drainage implant; N/A, Not available; GDI, glaucoma drainage implant.

Penetrating canaloplasty (PCP) is an available option to surgically treat PACG (20), and angle-closure glaucoma secondary to ICE syndrome (5), suggesting the feasibility of restoring the aqueous humor outflow in ICE through directly linking the posterior chamber and SC in a bleb-independent manner. Our previous study reported a successful treatment of PACG with extensive PAS by trabeculo-canalectomy, validating the feasibility of restoring the physiological outflow pathway in angle-closure eyes to control IOP (8). In our study, the mean IOP and the number of medications at each follow-up visit were significantly lower than baseline (Tables 2, 3 and Figure 2), confirming the high efficacy of bleb-independent trabeculo-canalectomy in treating GS-ICE via restoring the aqueous humor outflow.

After a precise localization of the SC, its outer wall and the adjacent TM were surgically removed during the trabeculo-canalectomy, leaving two cutting ends open into the aqueous humor (Figures 1c–e). This internal-filtrating procedure without the use of antimetabolites tightly sutured the scleral and conjunctival flaps, remarkably decreasing the risk of relevant complications (e.g., hypotony and overfiltration-associated shallow anterior chamber) (21). Additionally, postoperative bleb revisions by releasable sutures or needling were not necessary (22), greatly lowering the medical cost and improving the quality of life. Collectively, trabeculo-canalectomy increased the aqueous outflow through an anatomical route, without additionally adding postoperative interventions.

The long-term prognosis of GS-ICE is relatively poor due to the young onset age and a progressive pathology of ICE syndrome (e.g., PAS formation, endothelial proliferation, and bleb fibrosis) (18). A recent study proved the one-year efficacy and safety of retrocorneal membrane interception-enhanced PCP, a surgical procedure to create a barrier via suturing the cornea and iris at the drainage site, in treating open-angle or small-PAS GS-ICE, thus allowing a smooth filtration route by preventing the membrane proliferation (10). Instead of building up a barrier, we directly removed the outer wall of SC, adjacent TM, and a piece of iris tissue at the 12:00 position, remarkably simplifying surgical procedures and decreasing medical cost.

Notably, 2 years after surgery, visual acuity declined in GS-ICE patients (Table 2). We speculate this decline is related to the progression of cataract following trabeculectomy. Evidence has accumulated to show that trabeculectomy hastens the development of cataract (23, 24). In the study by Ron et al., the average time from trabeculectomy to cataract surgery was 26 months (23). Rahat et al. conducted an observational case series study of 177 patients who underwent a single trabeculectomy (25), finding that 77 (66%) showed cataract progression during the first year. Of these 77 patients, 63 (82%) had lens opacity in the posterior subcapsular region. However, the cause of cataract formation after trabeculectomy is not well understood and possibly due to altered aqueous humor dynamics affecting lens nutrition and metabolism (26, 27), which needs the clarification in future research.

Limitations existed in our study. First, this was a single-arm, small-scale, retrospective study lacking a control group of GS-ICE patients treated with conventional trabeculectomy or Ahmed valve implantation. Second, only Chinese patients with GS-ICE were retrospectively included. Third, the inclusion criteria in our study required a minimum follow-up of 2 years, which may exclude patients with poor follow-up, complications, or early failures, thereby overestimating success rate. Additionally, the duration of the endothelial membrane covering the fistula after trabeculo-canalectomy remains unclear. The study lacks OCT data of the optic disc in GS-ICE patients, which is a standard monitoring tool for these patients. In the future, long-term outcomes of trabeculo-canalectomy in GS-ICE require further exploration in large-scale cohorts across race groups.

## 5 Conclusion

GS-ICE patients treated with trabeculo-canalectomy present favorable two-year outcomes, including an effective IOP control, a reduction of anti-glaucoma medications, and a low risk of postoperative complications.

## Data Availability

The original contributions presented in the study are included in the article/supplementary material, further inquiries can be directed to the corresponding author/s.
